# The *SWEET14* sugar transporter mediates mycorrhizal symbiosis and carbon allocation in *Dendrobium officinale*

**DOI:** 10.1186/s12870-025-06443-8

**Published:** 2025-04-02

**Authors:** Liumin Li, Xueying Wang, Hua Li, Muhammad Moaaz Ali, Xiaobo Hu, Ralf Oelmuller, Ahmed Fathy Yousef, Abdulwahed Fahad Alrefaei, Jianfu Liu, Faxing Chen

**Affiliations:** 1 College of Horticulture, Fujian Agriculture and Forestry University, Fuzhou, 350002 Fujian China; 2 Institute of Horticultural Science and Engineering, Huaqiao University, Xiamen, 361021 Fujian China; 3 The School of Life and Health Sciences, Hainan University, Haikou, 570228 Hainan China; 4 Plant Physiology, Matthias Schleiden Institute, Friedrich-Schiller-University Jena, Dornburgerstr. 159, 07743 Jena, Germany; 5 Department of Horticulture, College of Agriculture, University of Al-Azhar (Assiut Branch), Assiut, 71524 Egypt; 6 National Key Laboratory for Germplasm Innovation & Utilization of Horticultural Crops, Zhengzhou Fruit Research Institute, Chinese Academy of Agricultural Sciences, Henan 450009 Zhengzhou, China; 7 Department of Zoology, College of Science, King Saud University, P.O. Box 2455, Riyadh, 11451 Saudi Arabia

**Keywords:** *SWEET* gene, *Dendrobium officinale*, Orchid mycorrhiza, Sugar metabolism, Carbon distribution

## Abstract

Orchid mycorrhizal (OM) fungi form mutualistic relationships with orchids, facilitating the uptake of minerals from the soil in exchange for sugars and lipids provided by the plant. In this study, we explored the role of the *DoSWEET14* sugar transporter in *Dendrobium officinale* during OM symbiosis. Transcriptome sequencing revealed significant changes in gene expression in mycorrhizal roots, particularly the upregulation of *DoSWEET14*. Subcellular localization studies confirmed that *DoSWEET14* is localized to the plasma membrane, suggesting its involvement in nutrient exchange between the orchid and OM fungi. Complementation assays using a yeast strain deficient in hexose transport demonstrated that *DoSWEET14* has broad substrate specificity, efficiently transporting multiple monosaccharides. Additionally, overexpression of *DoSWEET14* in *Arabidopsis* increased sugar content without affecting biomass, supporting its role in carbohydrate transport and storage during symbiosis. These findings suggest that *DoSWEET14* plays a key role in regulating carbon allocation and stabilizing the mutualistic relationship between *Dendrobium officinale* and OM fungi.

**Supplementary Information:**

The online version contains supplementary material available at 10.1186/s12870-025-06443-8.

## Introduction

Orchids, like many terrestrial plants, often form a symbiotic relationship with specific fungi, known as orchid mycorrhiza (OM) [1]. This mutually beneficial partnership enhances the orchid’s ability to access essential mineral nutrients, particularly phosphorus and nitrogen. In return, the orchid transfers part of its synthesized organic carbon, such as carbohydrates, to the fungus, providing it with nutrition [2]. The fungus utilizes this organic carbon as an energy source to meet its energy requirements [3].

The orchid-fungal symbiosis is unique compared to other mycorrhizal associations like ectomycorrhiza and arbuscular mycorrhiza, primarily due to the orchid’s distinct life cycle and diverse trophic strategies [4]. During the early stages of seed germination and seedling development, orchids rely on the mycorrhizal fungus for essential nutrients. The fungus aids in the uptake of minerals and possibly provides organic compounds while receiving limited or no direct nutritional resources from the orchids [5]. This phase of the symbiotic relationship is often referred to as the “fungus-dependent” or “mycoheterotrophic” stage [6].

After infecting the plant, OM fungi form mycorrhizal structures and develop dense rolls of mycelium called pelotons in the cortical cells of the embryo stalk. The digestion of these pelotons by the host plant is a significant mechanism for capturing fungus-derived compounds in OM [7]. According to previous research, Dearnaley and Cameron [8] summarized the bidirectional nutrient flow model of OM symbionts. The mycorrhizal fungus transfers nutrients including carbon (C), phosphorus (P), and nitrogen (N) across the interfacial matrix. Fungal nutrients like P, N, and C also pass to the orchid as the pelotons are digested [9]. As the orchid matures and becomes photosynthetic, the flow of carbon from the plant to the fungus is established, marking the transition from a heterotrophic juvenile to an autotrophic adult [10]. While the mechanisms of nutrient exchange in ectomycorrhizal and arbuscular mycorrhizal associations have been extensively studied, orchid mycorrhizal (OMF) interactions remain less understood, presenting a fascinating area for ongoing research.

There is growing evidence that the colonization of symbiotic fungi in plants creates new carbon reservoirs and significantly influences carbon allocation and sugar-related metabolic processes in both the above-ground and root tissues [11, 12]. For example, Zhao et al. [12] demonstrated that inoculation by “*Rhizophagus irregularis*” or “*Glomus aggregatum* with high arbuscular mycorrhizal” colonization increased sucrose, glucose, and fructose levels in both the above-ground parts and roots of soybean compared to uninoculated controls. In a previous investigation, Shachar-Hill et al. [13] employed isotope-labeled sugars alongside comprehensive nuclear magnetic resonance (NMR) spectroscopic analyses, demonstrating the direct transfer of glucose from the host plant to the fungus.

SWEET proteins, a family of bidirectional sugar transporters independent of proton pump activity, are predominantly located in the plasma membrane, tonoplast, or Golgi membrane [14, 15]. Additionally, various *SWEET* genes are transcriptionally activated by biotrophic bacteria and fungi, which modulate SWEET protein expression to extract carbon from the host plant [16, 17]. In *Solanum tuberosum* (potato) and *Glycine max* (soybean), specific SWEET transporter genes have shown upregulation during mycorrhizal symbiosis [12], indicating that certain SWEET family members may play a role in providing sugars to arbuscular mycorrhizal fungi. Manck-Götzenberger and Requena [18] also observed that the promoter regions of *SWEET* genes in potatoes were highly active near cells containing arbuscules, suggesting that SWEET proteins may facilitate sugar transport from the plant to these specialized fungal structures. In light of this, the current study aims to investigate the role of SWEET sugar transporters in the mycorrhizal symbiosis of *Dendrobium officinale*.

In brief, this study explored the molecular role of the *SWEET14* gene in the mutualistic relationship between *Dendrobium officinale* and the OM fungus during the colonization phase. The findings revealed that the expression of the *DoSWEET14* gene was significantly upregulated during mycorrhizal symbiosis, indicating increased gene activity in the presence of the OM fungus. This suggests that *DoSWEET14* plays a crucial role in the symbiotic interaction. Additionally, this research provides valuable insights into the specific interactions between *Dendrobium officinale* and the OM fungus, while also establishing a theoretical basis for understanding the regulatory mechanisms governing sugar transporter gene expression in the Orchidaceae family, particularly in green orchid species.

## Materials and methods

### Experimental materials

*Dendrobium officinale* was supplied by the Xiamen National Forest Seed Improvement Base, Xiamen City. *Cladosporium halotolerans* was obtained from the Microbial Culture Preservation Center in Beijing, and *Mycena dendrobii* was sourced from Beijing Baiou Bowei Biological. *Nicotiana benthamiana*, along with vectors pCAMBIA1302 and pDR96, are maintained in our laboratory. *Escherichia coli* DH5α and *Agrobacterium tumefaciens* GV3101 competent cells, as well as the FastPure Plasmid Mini Kit, were purchased from Novizan Biotechnology Co., LTD. The yeast mutant strain EBY.VW4000 was kindly provided by Professor Chen Qingxi of Fujian Agriculture and Forestry University.

### Mycorrhizal symbiotic culture

A batch of sterile tissue-cultured seedlings, all with similar growth characteristics (4–6 leaves, plant height over 3 cm), were selected as the experimental seedlings for 7–10 days. *Mycena dendrobii* and *Cladosporium halotolerans* strains were inoculated into PDB liquid medium and incubated with shaking at 28 °C for 7–10 days. The tissue-cultured seedlings were transplanted into an autoclaved substrate (121 °C for 2 h), with 8 seedlings planted in each cavity dish. For each treatment, 5 mL of the fungal solution was applied to the exposed roots, while the control group received 5 mL of sterile water. After inoculation, the roots were covered with the substrate to ensure root stability and a consistent growth environment. Each treatment was replicated across 10 cavity dishes, with three biological replicates, totaling 30 cavity dishes per treatment.

### RNA sequencing

The root cDNA libraries of uninoculated (NM), *Mycena dendrobii*-inoculated (Md), and *Cladosporium halotolerans*-inoculated (Ch) *Dendrobium officinale* were sequenced using the BGISEQ-500 platform (NOO Zhiyuan, Shanghai, China). Differential gene expression was considered significant with an absolute Log_2_ fold change ≥ 1.0 and a probability ≥ 0.8. To identify functionally important gene sets in OM roots, we performed functional enrichment analysis of differentially expressed genes (DEGs). Gene ontology (GO) classification and KEGG pathway enrichment analyses were carried out using the bioinformatics tool TBtools, while ggplot2 was employed for data visualization.

### Validation of DEGs by qRT-PCR

Total RNA was extracted from the roots, stems, and leaves of *Dendrobium officinale* using the RNAprep Pure Polysaccharide Polyphenol Plant Total RNA Extraction Kit (Tiangen Biochemical Technology Co., LTD.). cDNA synthesis was performed using RNA as a template with the Reverse Transcription Kit (Novozan Biotechnology Co., LTD.). Quantitative real-time PCR (qRT-PCR) was conducted using the 2× SYBR Green Pro Taq HS Premix Kit (Ecoray Biotechnology Co., LTD.) on a fluorescence-based quantitative PCR instrument to analyze the expression patterns of selected genes. Specific primers were designed for four sugar transporter genes (*DoMST4*, *DoMST5*, *DoSWEET14*, and *DoSWEET4*) based on their sequences. Relative expression levels of these target genes across different tissues were measured using cDNA and the designed primers. Additionally, fungal 18S rRNA gene expression was analyzed to confirm Dendrobium mycorrhizal colonization. The qPCR reaction conditions were as follows: initial denaturation at 95°C for 5 seconds, followed by 40 cycles of 95°C for 5 seconds, 55°C for 30 seconds, and 72°C for 30 seconds. The melting curve analysis involved 95°C for 5 seconds, 60°C for 30 seconds, and 95°C for 5 seconds. Gene expression was normalized to the 18S rRNA gene using the 2^−ΔΔCT^ method, with PCR amplification efficiencies ranging between 90% and 110%. All qRT-PCR experiments were performed in triplicate. Primers used for the qRT-PCR analysis are listed in Table S1.

### Agrobacterium rhizogenes-mediated transformation

*DoSWEET14a* and *DoSWEET14b* were PCR-amplified from *Dendrobium officinale* cDNA using primers containing KpnI and EcoRI restriction sites, and then inserted into the plant binary expression vector pCAMBIA1302. The resulting *DoSWEET14a-pCAMBIA1302* and *DoSWEET14b-pCAMBIA1302* constructs were verified by Sanger sequencing. Various vectors were transformed into *Agrobacterium tumefaciens* GV3101 (pSoup-p19) for subsequent infection of *Nicotiana benthamiana* plants. The pSoup-p19 plasmid contains the pSoup helper plasmid and the P19 protein from the tomato bushy stunt virus, which is known to inhibit RNA silencing in plants, enhancing the stability and expression of heterologous genes [19].

The transformed *Agrobacterium* cultures were grown to an optical density at 600 nm (OD600) of approximately 0.6. Once the desired OD600 was reached, the bacterial cultures were collected by centrifugation to concentrate the cells. The bacterial pellet was resuspended in an infection solution (10 mM MgCl2, 10 mM MES, and 100 μM acetosyringone). This bacterial suspension, containing the plasmid of interest (pCAMBIA1302-DoSWEET14) and potentially a plasma membrane marker protein, was infiltrated into the lower surface of the apical three leaves of *Nicotiana benthamiana* using a sterile syringe.

After infiltration, the plants were incubated in darkness for 48 hours. Two days after being transferred to low-light conditions, the subcellular localization of the gene of interest and the marker protein was examined using a laser confocal microscope. GFP fluorescence was excited at 488 nm, and mCherry fluorescence was excited at 561 nm.

### Yeast growth assays

The *DoSWEET*s were PCR-amplified from *Dendrobium officinale* cDNA using primers with PstI and XbaI restriction sites, and the resulting products were cloned into the yeast expression vector pDR196. The final constructs for *DoSWEET14a* and *DoSWEET14b* were confirmed by Sanger sequencing. All vectors were introduced into the yeast hexose transporter mutant strain EBY.VW4000 using the PEG/LiAc-mediated transformation method [20]. After transformation, yeast transformants were cultured on selective dropout (SD, -URA3) medium containing 2% maltose as the carbon source for 2–3 days at 28°C. The presence of the constructs in the yeast transformants was confirmed by plasmid isolation and resequencing.

For complementation growth assays, the transformants were grown overnight in YPDA liquid medium containing 2% maltose, washed twice with sterile water, and resuspended to an OD600 of 0.2. Serial dilutions (10x, 100x, 1000x, and 10,000x) were then plated onto SD (-URA3) media containing either 2% maltose (control) or 2% of various sugars: glucose, fructose, mannose, galactose, rhamnose, xylose, arabinose, or sucrose. Growth was recorded after 2–4 days of incubation at 28°C. 

### Genetic transformation of Arabidopsis

*DoSWEET* genes were PCR-amplified from *Dendrobium officinale* cDNA using primers containing KpnI and EcoRI restriction sites, and then inserted into the plant binary expression vector pCAMBIA1302. The resulting *DoSWEET14-pCAMBIA1302* constructs were confirmed by Sanger sequencing. These vectors were transformed into *Agrobacterium tumefaciens* GV3101 for subsequent infection of wild-type Arabidopsis Col-0 plants. After a 24-hour dark treatment, the seeds were cultured under normal growth conditions until harvest. Seeds from the T1 generation underwent vernalization, a cold treatment at 4 °C, before germination and seedling growth.

Two to three weeks later, a 0.01% Basta solution was sprayed onto the leaves of the seedlings. Resistant seedlings were transplanted into new pots for further growth. After sufficient growth, total RNA was extracted using the RNAprep Pure Polysaccharide Polyphenol Plant Total RNA Extraction Kit. Independent transformation lines were identified based on PCR results. From the T2 generation lines, at least three homozygous lines were selected, which were likely considered stable. These homozygous lines were chosen for further experiments, such as phenotypic analysis, functional studies, or other genetic investigations.

### Analysis of soluble sugars

The chloroform-methanol technique was used to extract soluble sugars from roots and shoots [21]. The amount of soluble sugar was given as milligrams (mg/g DW) per gram of dry weight.

### Statistical analyses

Data were analyzed using Origin 2021, and statistical significance (*P* ≤ 0.05) was assessed with a two-way ANOVA, followed by Duncan’s multiple range test for mean separation in SPSS (SAS Institute Inc., Cary, NC). Statistically significant differences between treatment groups (*P* ≤ 0.05) are indicated by lowercase letters. Results are expressed as mean ± standard deviation, based on three biological replicates.

## Results

### Overview of the gene expression profiles

**Fig. 1:**
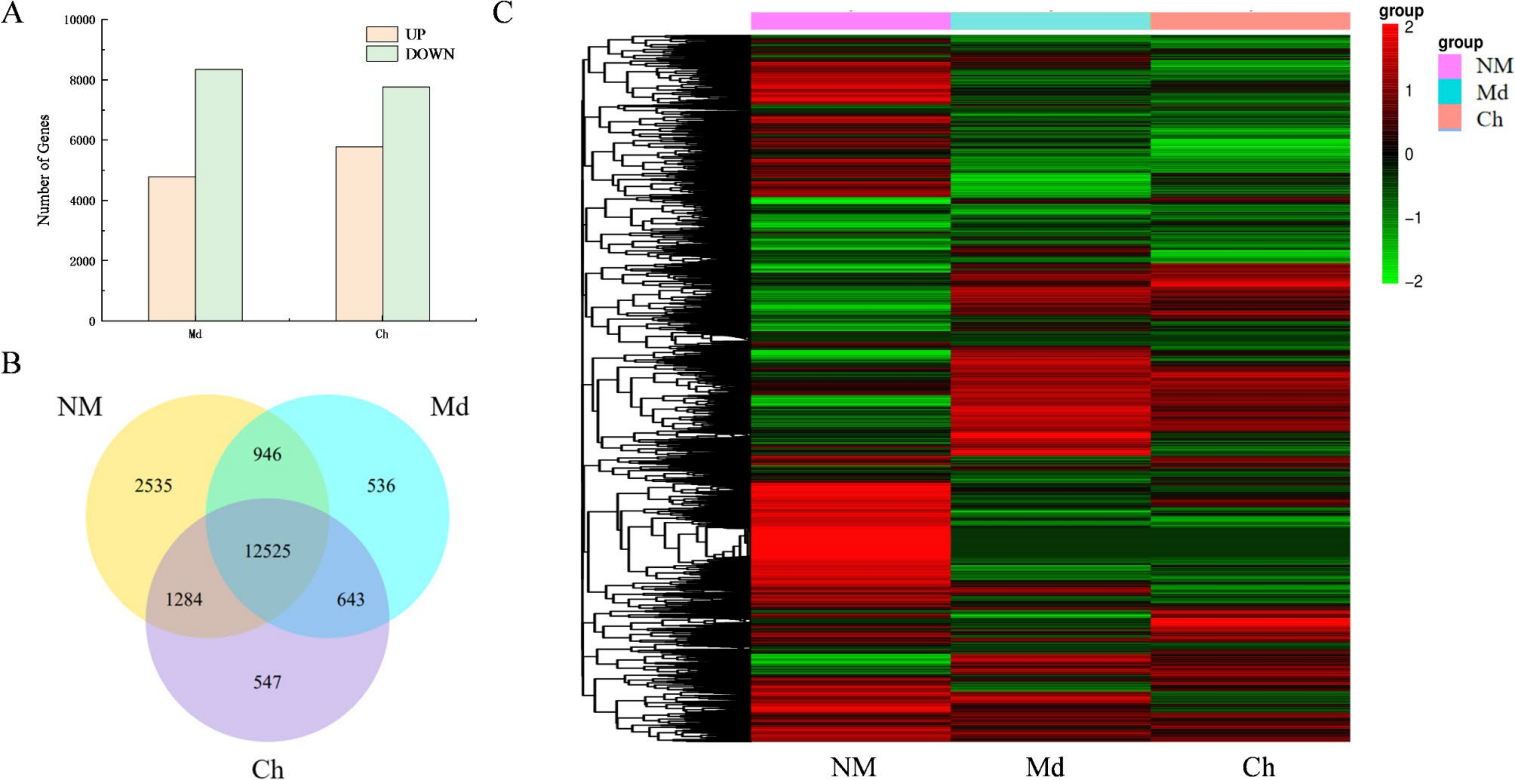
Number of DEGs (**A**), Venn diagrams (**B**), and cluster analysis of DEGs in *Dendrobium officinale* roots in response to *Mycena dendrobii* (Md) and *Cladosporium halotolerans* (Ch) inoculation

To assess the effects of different mycorrhizal inoculations on the transcriptional response of *Dendrobium officinale* roots, we analyzed the expression profiles of transcripts in NM and OM roots. Differential expression analysis was performed using the DESeq2 package to compare gene expression between NM and OM-inoculated roots, with a Q-value (adjusted *P*-value) threshold of ≤ 0.05 to control the false discovery rate. *Mycena dendrobii* (Md) and *Cladosporium halotolerans* (Ch) treatments identified a total of 26,662 DEGs. Of these, 10,552 genes were up-regulated and 16,110 were down-regulated. According to the Venn diagram, only 643 genes were differentially expressed in both Md and Ch treatments, suggesting that a relatively small subset of genes responded similarly to both inoculations, while the majority exhibited distinct transcriptional responses (Fig. 1).

### Validation of DEGs by qRT-PCR

**Fig. 2:**
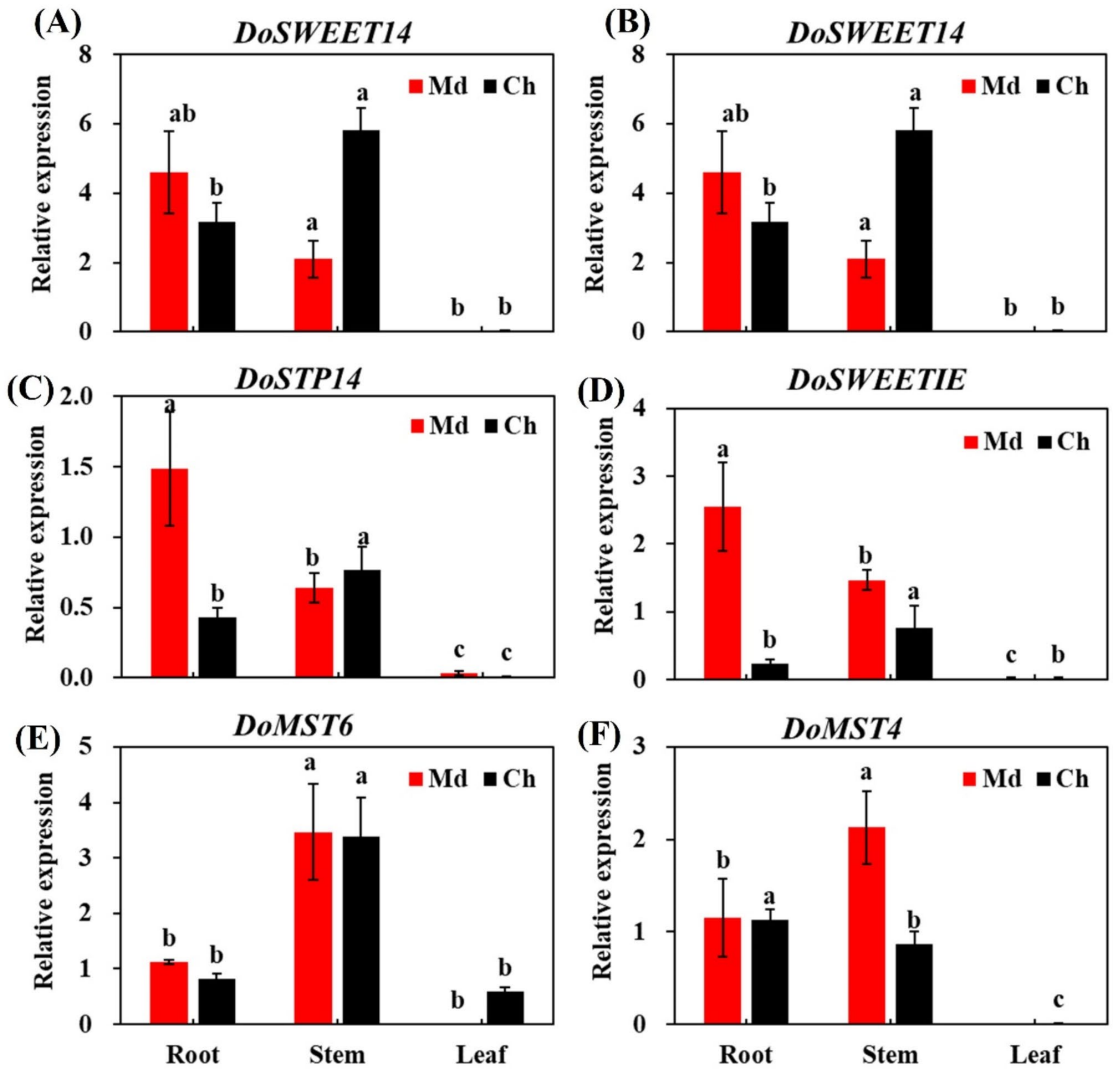
qRT-PCR analysis of specific gene expression levels in *Dendrobium officinale* tissues. Results are presented as mean ± standard deviation from three biological replicates, with significant differences between groups or treatments (*P* ≤ 0.05) indicated by lowercase letters, as determined by Duncan’s multiple range test

The qRT-PCR results were compared with the differential expression profiles obtained from RNA-Seq data (Fig. 2). *DoSWEET14*, *DoMST4*, and *DoMST6* genes exhibited significantly higher expression levels in stems compared to roots and leaves, indicating tissue-specific expression patterns. In contrast, *DoSWEET4* showed the highest expression in roots, followed by stems. After mycorrhizal fungi inoculation, the transcription levels of these genes increased significantly in both roots and stems, suggesting their responsiveness to mycorrhizal associations and upregulation in response to symbiotic fungi. The consistency between the qRT-PCR results and the RNA-Seq data further validates the reliability of the sequencing findings and supports the study’s conclusions.

### GO and KEGG enrichment analysis of DEGs

The GO functional classification of DEGs in *Dendrobium officinale* mycorrhiza revealed 10,552 up-regulated and 16,110 down-regulated genes in Md and Ch mycorrhiza. These genes were divided into three groups: molecular functions, cellular components, and biological activities. The DEGs in the *Dendrobium* mycorrhiza (OM) were predominantly associated with molecular functions, particularly in processes related to the cell cortex, transport activity, microtubule binding, and tubulin binding (Figure S1).

**Fig. 3:**
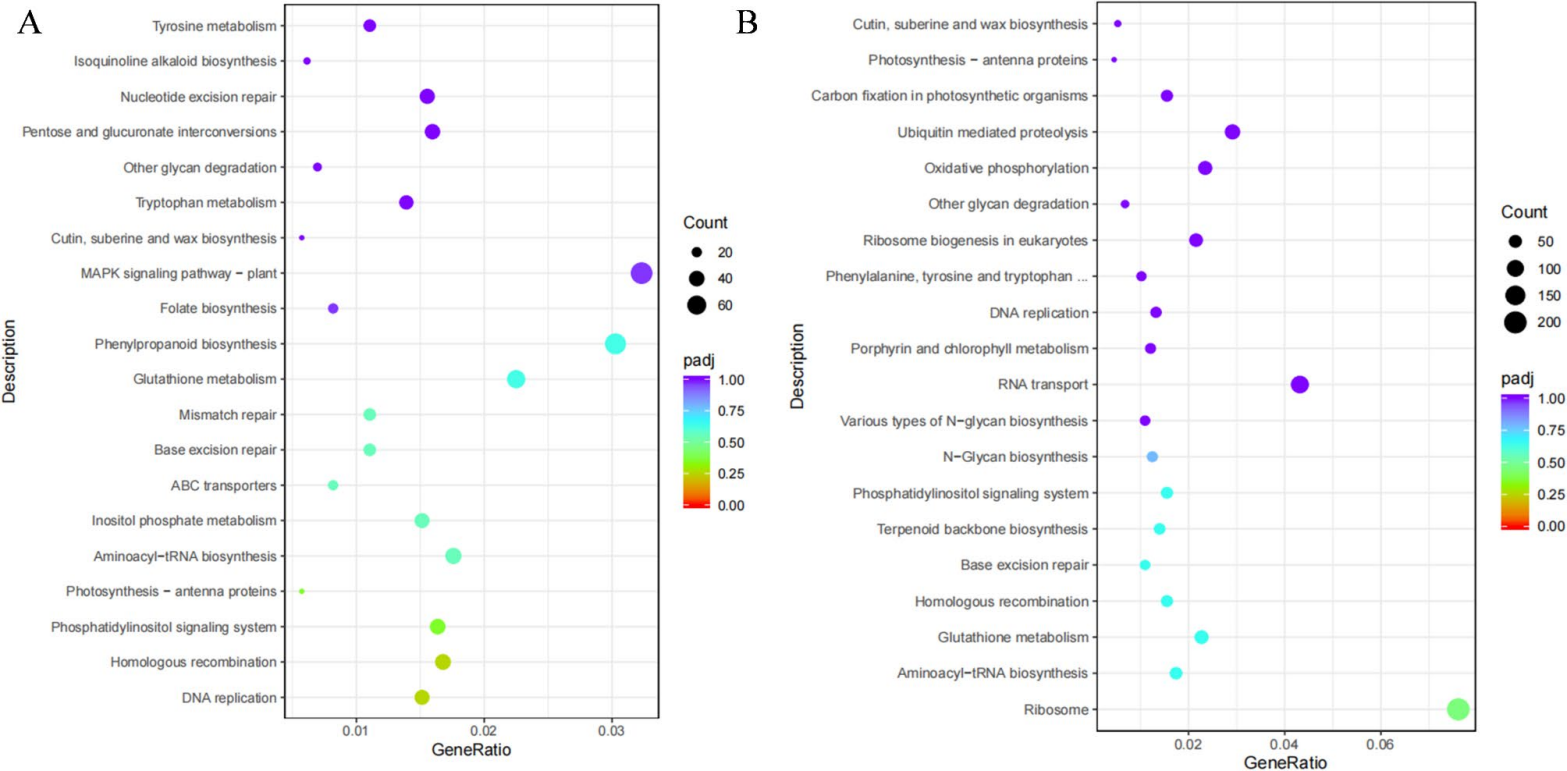
KEGG enrichment pathway analysis of up-regulated genes in Orchidaceae mycorrhiza inoculated with *M. dendrobii* (**A**) and *C. halotolerans* (**B**). The vertical axis denotes the enriched pathways, whereas the horizontal axis illustrates the enrichment factors. The dimensions of each point represent the quantity of DEGs associated with the pathway, while the color denotes the *P*-value. Pathways exhibiting a *P*-value less than 0.05 were classified as significantly enriched

Additionally, the KEGG database was used to further characterize DEGs involved in metabolic pathways. Figure 3A displays a scatter plot that depicts the enriched pathways. Twenty KEGG-enriched pathways in total were found to have higher DEG levels in all OM roots as compared to NM roots. Interestingly, at least five highly enriched KEGG pathways were strongly linked to sugar metabolism, including the pentose phosphate route, carbon fixation in photosynthetic organisms, fructose and mannose metabolism, glycolysis/gluconeogenesis, and glycan synthesis and metabolism (Fig. 3B).

### The *DoSWEET14* localizes to the peri-arbuscular membrane

**Fig. 4:**
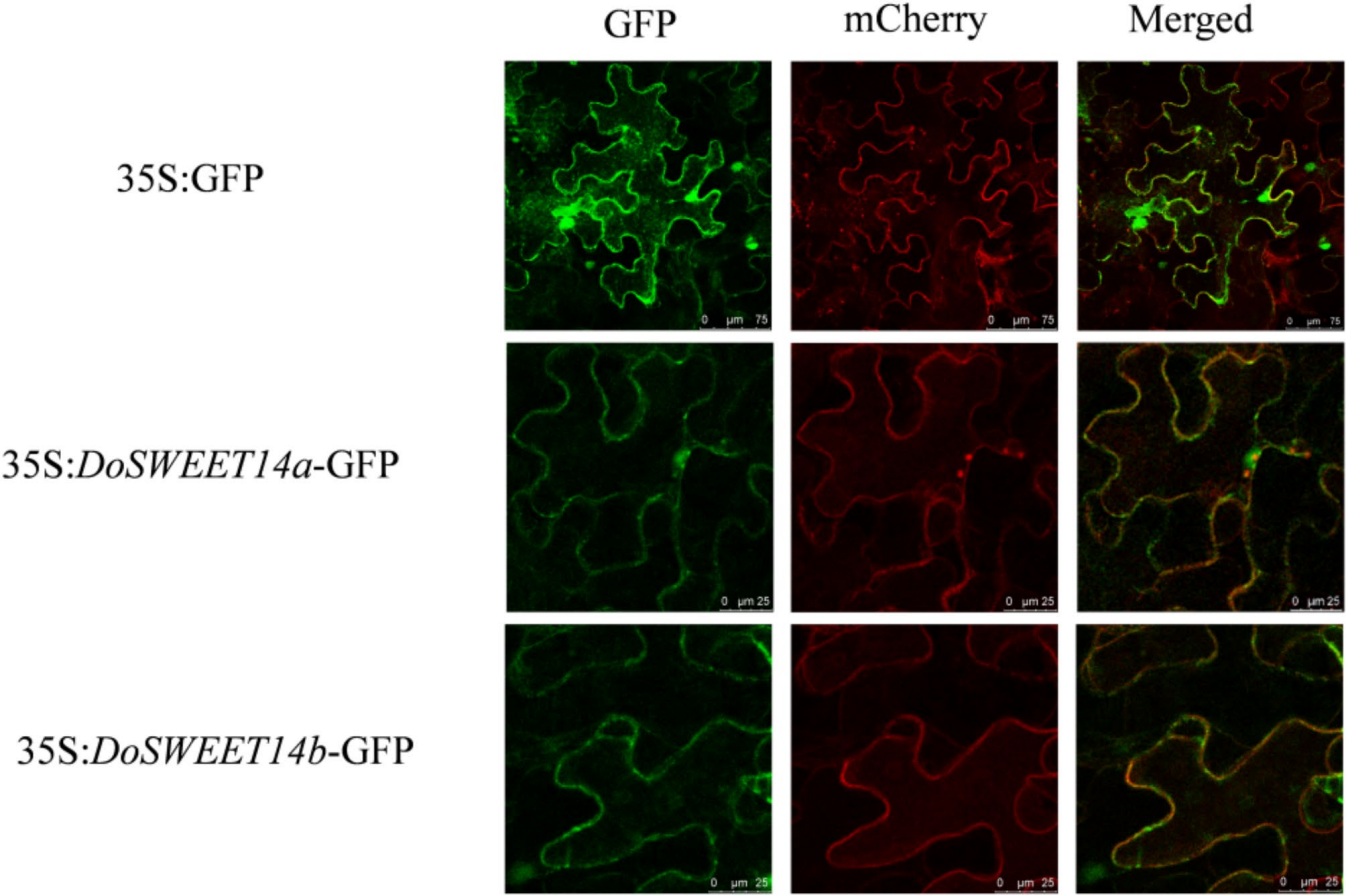
Subcellular localization of the DoSWEET14-GFP fusion protein in *Dendrobium officinale* cells

A fusion protein construct was generated by linking the DoSWEET14 protein to a green fluorescent protein (GFP) tag. Along with the DoSWEET14-GFP fusion protein, a plasma membrane marker, AtPIP2A: mCherry, was co-expressed. This marker, tagged with the red fluorescent protein mCherry, is known to localize specifically to the plasma membrane, aiding in the verification of DoSWEET14 subcellular localization. An empty pCAMBIA1302-GFP vector served as the control. Confocal imaging results revealed that the pCAMBIA1302-DoSWEET14-GFP fusion protein localized to the plasma membrane (Fig. 4). These findings suggest that DoSWEET14 localization in the plasma membrane may play a key role in regulating mycorrhizal symbiosis in *Dendrobium officinale*.

### DoSWEET14 has a broad substrate specificity and monosaccharide transport profile

**Fig. 5:**
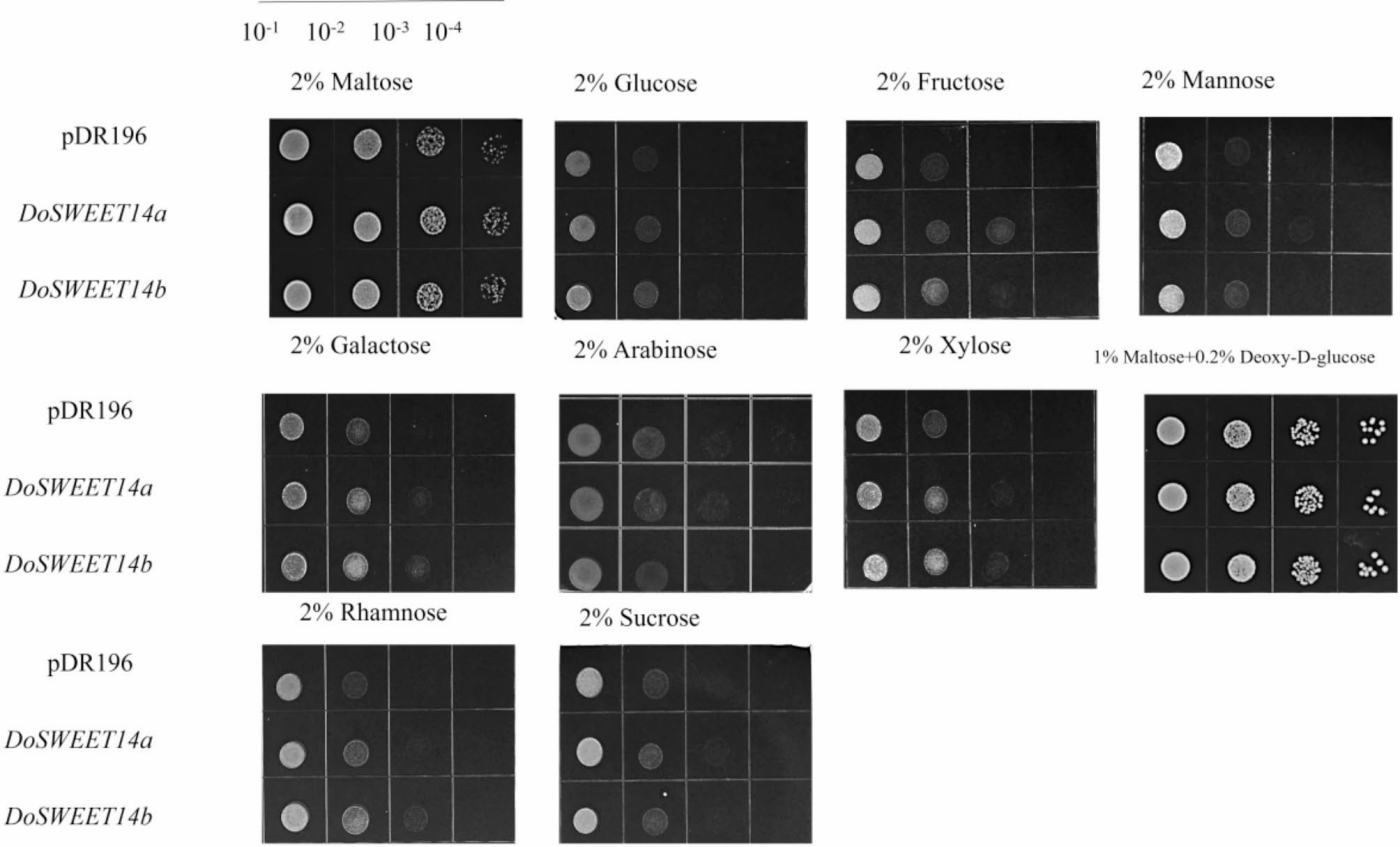
Analysis of the monosaccharide transport activity of *DoSWEET14*. Yeast strain EBY.VW4000, which lacks 20 endogenous monosaccharide transporter genes, was transformed with the *DoSWEET14* gene under the control of the Adh1 promoter. Growth assays were conducted to assess the transport activity of *DoSWEET14* on various sugars

We further investigated the sugar transport activity and substrate specificity of the DoSWEET14 protein. The *DoSWEET14* gene was inserted into the pDR196 plasmid, placing it under the control of the Adh1 promoter. The yeast strain EBY.VW4000, which lacks 20 endogenous monosaccharide transporter genes, was used for this experiment. This strain cannot grow on media containing hexose sugars (e.g., glucose and sucrose) but can grow on maltose-containing media. No significant difference in growth was observed between Adh1-*DoSWEET14* and Adh1-E yeast strains on maltose-containing media. However, the expression of *DoSWEET14* under specific conditions enhanced the yeast’s ability to grow on certain sugars, including fructose, galactose, mannose, rhamnose, and sucrose. There was no significant effect on the utilization of glucose or xylose, as both strains exhibited similar growth on media containing these sugars (Fig. 5). We hypothesize that the regulation of *DoSWEET14* in *Dendrobium officinale* by OM fungi may influence carbon partitioning among cortical cells, allowing arbuscule-containing cells to accumulate sugars to support the increased metabolic demands of the symbiotic fungus.

### Effect of inoculum treatments on sugar content

**Fig. 6:**
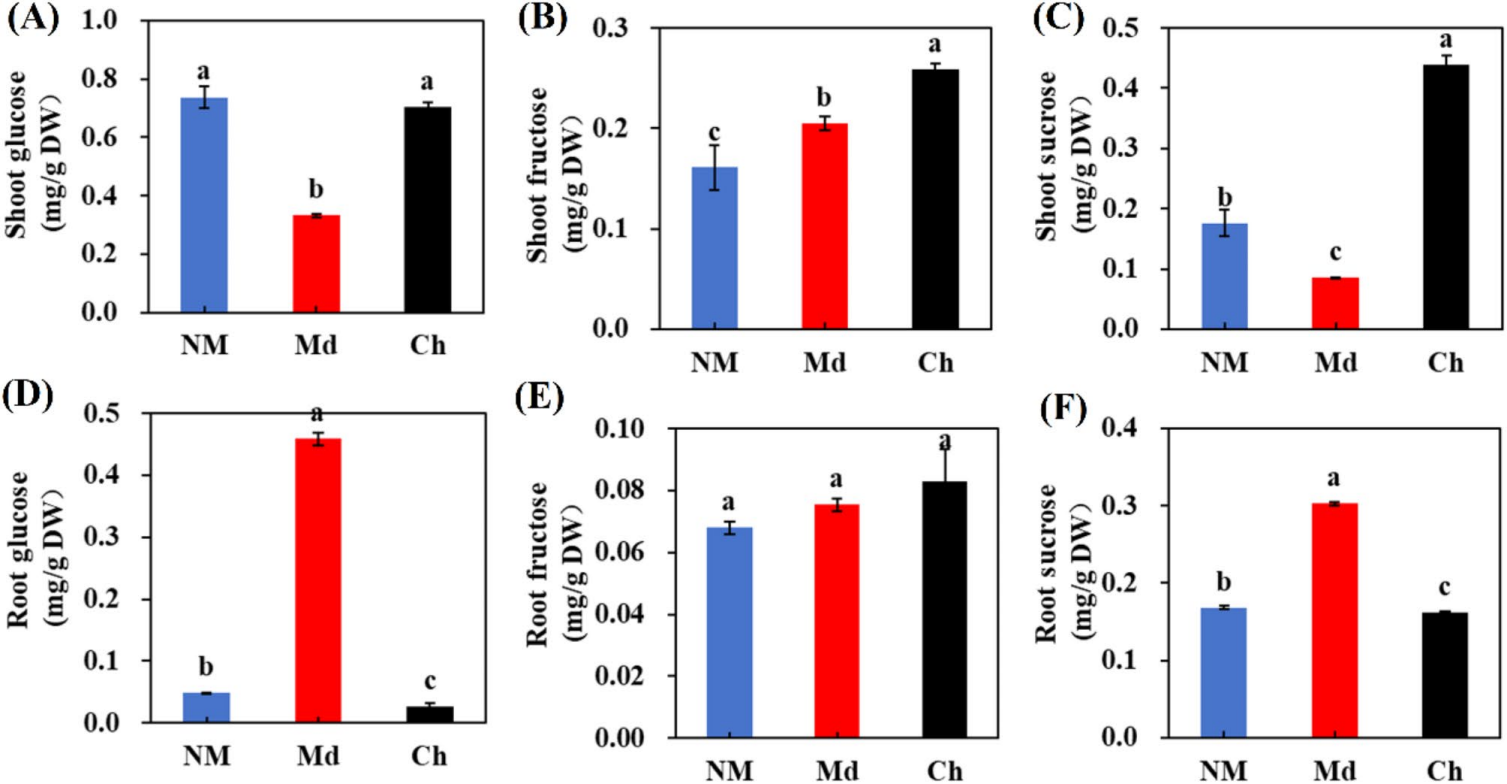
Effect of mycorrhizal treatment on sugar content of *Dendrobium officinale*. Results are presented as the mean ± standard deviation of three biological replicates, with lowercase letters indicating significant differences between treatments (*P* ≤ 0.05) following Duncan’s multiple range test

**Fig. 7:**
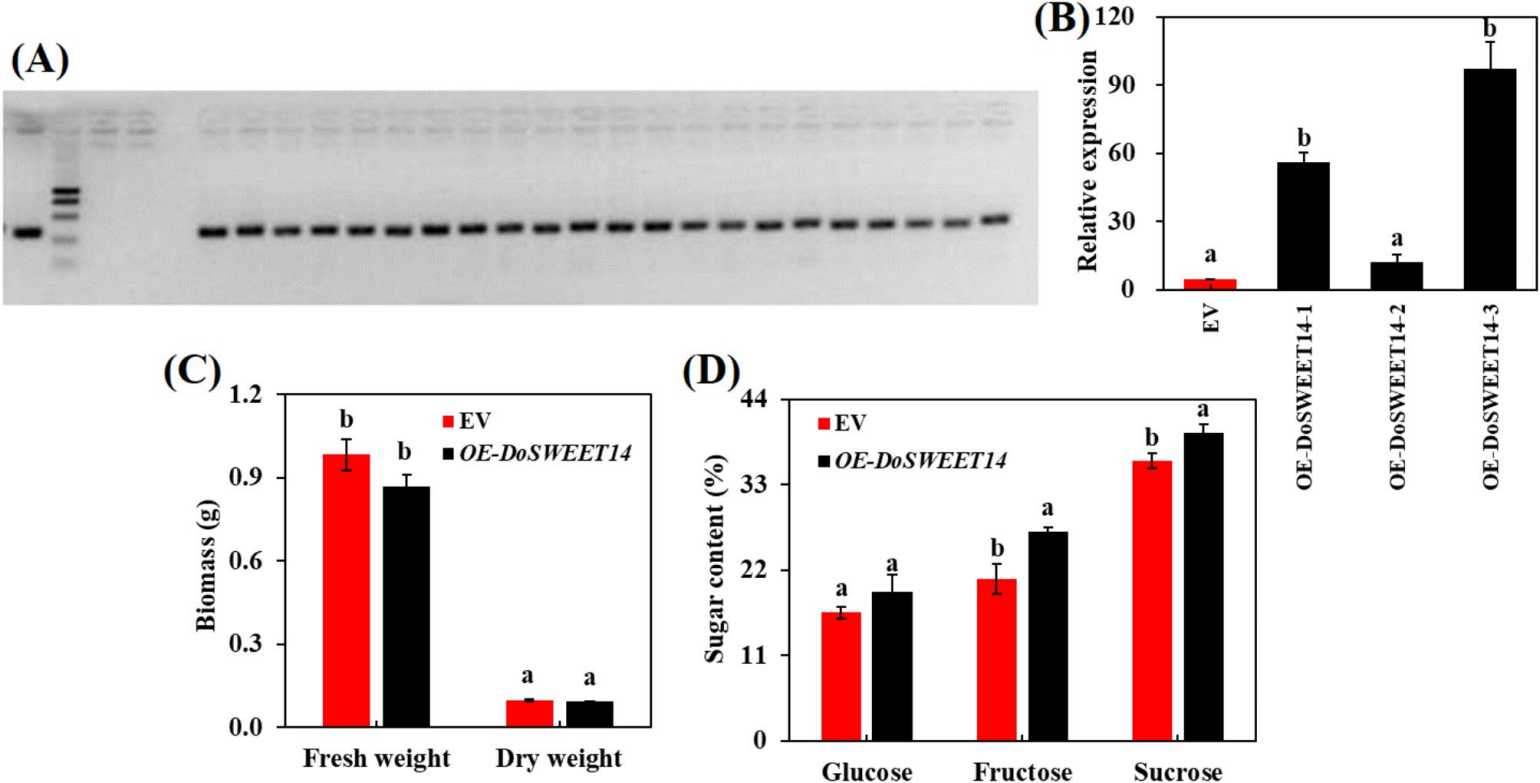
Overexpression of *DoSWEET14* in *Arabidopsis*. (**A**) PCR verification of *DoSWEET14* insertion. (**B**) RT-qPCR analysis showing the relative expression levels of *DoSWEET14*. (**C**) Biomass comparison between *DoSWEET14*-overexpressing plants and wild-type plants. (**D**) Sugar content analysis, including glucose, fructose, and sucrose levels. Results are presented as the mean ± standard deviation of three biological replicates, with lowercase letters indicating significant differences between treatments (*P* ≤ 0.05) following Duncan’s multiple range test

Regardless of the inoculation status of the plants, the roots showed much greater amounts of fructose and glucose than the shoots (Fig. 6). In comparison to plants inoculated with Ga-L or non-mycorrhizal (NM) plants, plants colonized by Ri and Ga-H showed considerably greater amounts of glucose, fructose, and sucrose in both shoots and roots (Fig. 7). These results suggest that microbial colonization influences carbon distribution within plant tissues, likely mediated by sugar transporters.

### Overexpression of *DoSWEET14*

The expression levels of all other *SWEET* genes were analyzed in *OE-DoSWEET14* mutants and wild-type *Col-1* plants using RT-qPCR (Fig. 7). The results confirmed that *DoSWEET14* expression was significantly higher in the mutants compared to the empty vector (EV) controls. Additionally, overexpression of *DoSWEET14* did not result in a significant difference in biomass compared to wild-type plants. However, it notably altered carbon allocation in *Arabidopsis*, with the overexpressing plants showing significantly higher levels of glucose, fructose, and sucrose compared to the wild type. These findings align with previous results on the role of *DoSWEET14* in sugar transport and carbon distribution, suggesting that *DoSWEET14* may further regulate carbon allocation by influencing sugar metabolism in plants.

## Discussion

SWEET transporters are integral to various physiological processes beyond just sugar transport. They are involved in phloem loading, nectar secretion, seed filling, and responses to both biotic and abiotic stresses [22, 23]. The ability of SWEETs to function as bidirectional uniporters allows them to facilitate sugar movement across membranes without relying on proton gradients [24].

Sugar transport is a crucial interface where plants and their symbiotic microbes interact, each aiming to optimize carbon allocation for their own benefit, with SWEET transporters emerging as key mediators in this process [25]. The interaction between plants and their microbial partners often involves competition for sugars. As highlighted by recent studies, both plants and microbes utilize monosaccharide transporters alongside SWEETs to secure necessary nutrients during their interactions [26]. Previous studies have demonstrated that SWEET family members exhibit dual sugar efflux and influx activities across various plant species [27]. In this study, we provide the first evidence that orchid mycorrhizal (OM) symbiosis induces significant transcriptomic reprogramming in *Dendrobium officinale* roots, particularly in genes involved in sugar and lipid metabolism and transport. Our results show that *DoSWEET14* is strongly upregulated in root cortex cells colonized by OM fungi, with localization to the peri-arbuscular membrane surrounding the arbuscules.

This finding aligns with previous research in potato, where several *SWEET* genes, including those from clade II and clade III (sucrose transporters), were induced in arbuscule-containing cells during mycorrhization [18, 28]. Similarly, *MtSWEET1b* in *Medicago truncatula* is highly expressed in root nodules and other tissues involved in symbiosis, supporting its role in both arbuscular mycorrhizal (AM) and rhizobial symbioses [29]. These findings suggest that while the regulation of *SWEET* genes may vary across plant species, their function in carbon partitioning during symbiosis remains crucial. We hypothesize that this regulation could influence the symbiotic efficiency of different plant-fungal pairings.

Our data indicate that OM symbiosis substantially alters the transcription of genes involved in sugar and lipid metabolism in *Dendrobium officinale* roots. Colonization by OM fungi introduces an additional carbon sink, leading to increased allocation of photosynthates to root systems [30, 31]. The higher expression of *DoSWEET14* in arbuscule-containing cells suggests its role in facilitating carbon transfer to OM fungi, similar to *MtSWEET1b* in AM symbiosis, which has been linked to sugar export to the fungal partner [32]. In our study, the increased levels of glucose, fructose, and sucrose in plants colonized by Md and Ch further support this role. This redistribution of carbon may be driven by *DoSWEET14*, allowing host plants to support the heightened metabolic demands of the symbiotic fungi. The transcriptomic changes observed during OM symbiosis suggest that there are extensive regulatory networks at play. For example, the upregulation of *DoSWEET14* may not only facilitate sugar transfer but also indicate a broader metabolic reprogramming necessary for sustaining the symbiotic relationship [33].

The diversity within the SWEET family is notable. Clades I and II primarily transport hexoses like glucose and fructose, while clade III is more specialized for sucrose transport [22, 24]. Interestingly, while most SWEET transporters are known to specialize in transporting specific types of sugars, some have broader substrate specificity, including both mono- and disaccharides [34], and others have even been shown to transport non-sugar substrates such as gibberellins [35, 36]. In our study, complementation assays using the hexose transport-deficient yeast strain EBY.VW4000 confirmed that *DoSWEET14* has a broad monosaccharide transport profile, further demonstrating its potential versatility in sugar transport.

In conclusion, our findings suggest that SWEET transporters, particularly *DoSWEET14*, play a significant role in stabilizing OM symbiosis in *Dendrobium officinale* by regulating carbon allocation, potentially enhancing the symbiotic relationship through efficient sugar transport. Further research into the functional roles of specific SWEET transporters within various plant-microbe systems could provide insights into optimizing agricultural practices. Understanding how these transporters can be manipulated through genetic engineering or breeding strategies may enhance crop resilience against pathogens while improving nutrient allocation efficiency.

## Conclusions

In this study, RNA-seq analysis revealed significant upregulation of sugar transporter genes, particularly *DoSWEET14*, in *Dendrobium officinale* roots colonized by orchid mycorrhizal fungi. Our findings highlight *DoSWEET14* as a key player in sugar allocation, stabilizing the symbiotic relationship. Subcellular localization confirmed its presence in the plasma membrane, while yeast complementation assays demonstrated its broad substrate specificity. Overexpression in *Arabidopsis* further supported its role in carbohydrate transport without affecting biomass. These results provide new insights into the function of SWEET transporters in regulating carbon allocation during mycorrhizal symbiosis. Future studies could explore genetic modifications to enhance beneficial symbioses, improving plant growth and stress resilience in horticultural and agricultural systems.

## Electronic supplementary material

Below is the link to the electronic supplementary material.

Supplementary Material 1

## Data Availability

The RNA-seq data generated and analyzed during the current study are publicly available in the NCBI Sequence Read Archive (SRA) under the accession number PRJNA1192056.
