# Supplementary material for: The SWEET14 sugar transporter mediates mycorrhizal symbiosis and carbon allocation in Dendrobium officinale
Source: BMC Plant Biol. 2025 Apr 2;25:416. doi: 10.1186/s12870-025-06443-8 (PMC11963625; doi:10.1186/s12870-025-06443-8)
Supplement: Supplementary file 1 — Supplementary Material 1 [file 12870_2025_6443_MOESM1_ESM.docx]

**Supplementary material**

**Supplementary Table S1. Primers for differential gene RT-qPCR**

| Gene | primer sequence （5´-3´） |
| --- | --- |
| *DoSWEET14-F* | GATGGATCTGTGTTGGCTTCTC |
| *DoSWEET14-R* | CCACACTCTTGGTTCGTATGAC |
| *DoSWEET4-F* | TCTTGGTGACTTGGCATTCG |
| *DoSWEET4-R* | GACACATAGGATACCGACAACA |
| *DoMST3-F* | TGATGGATTGAAGGTGGAGTGA |
| *DoMST3-R* | ACTGATTCGTGCTCGTATCTTG |
| *DoMST6-F* | ATGCTCGCTTGCCAGATTAC |
| *DoMST6-R* | TGAAGACCACTACGGTGAAGG |
| *DoSWEETIE-F* | ACGGAGAAGTGAGGTGAAGAG |
| *DoSWEETIE-R* | AGGAGCAGAAGCGACGATAG |

**Supplementary Figure S1. GO analysis of DEGs in the roots of *Dendrobium officinale***

**注：A、NM Vs Md；B、NM Vs Ch.**

**Note: A、NM Vs Md；B、NM Vs Ch.**
